# Why do people choose not to take part in screening? Qualitative interview study of atrial fibrillation screening nonparticipation

**DOI:** 10.1111/hex.13819

**Published:** 2023-07-14

**Authors:** Sarah Hoare, Gwilym P. A. Thomas, Alison Powell, Natalie Armstrong, Jonathan Mant, Jenni Burt

**Affiliations:** ^1^ The Healthcare Improvement Studies Institute (THIS Institute), Department of Public Health and Primary Care University of Cambridge Cambridge UK; ^2^ The Guildhall and Barrow Surgery Bury St Edmunds UK; ^3^ Primary Care Unit, Department of Public Health and Primary Care Strangeways Research Laboratory, University of Cambridge School of Clinical Medicine University of Cambridge Cambridge UK; ^4^ SAPPHIRE Research Group, Department of Population Health Sciences University of Leicester Leicester UK

**Keywords:** atrial fibrillation, declining to participate, interviews, qualitative, screening, sociology, United Kingdom

## Abstract

**Introduction:**

While screening uptake is variable, many individuals feel they ‘ought’ to participate in screening programmes to aid the detection of conditions amenable to early treatment. Those not taking part in screening are often presented as either hindered by practical or social barriers or personally at fault. Why some people *choose* not to participate receives less consideration.

**Methods:**

We explored screening nonparticipation by examining the accounts of participants who chose not to participate in screening offered by a national research trial of atrial fibrillation (AF) screening in England (SAFER: Screening for Atrial Fibrillation with ECG to Reduce stroke). AF is a heart arrhythmia that increases in prevalence with age and increases the risk of stroke. Systematic screening for AF is not a nationally adopted programme within the United Kingdom; it provides a unique opportunity to explore screening nonparticipation outside of the norms and values attached to existing population‐based screening programmes. We interviewed people aged over 65 (*n* = 50) who declined an invitation from SAFER and analysed their accounts thematically.

**Results:**

Beyond practical reasons for nonparticipation, interviewees challenged the utility of identifying and managing AF earlier. Many questioned the benefits of screening at their age. The trial's presentation of the screening as research made it feel voluntary—something they could legitimately decline.

**Conclusion:**

Nonparticipants were not resistant to engaging in health‐promoting behaviours, uninformed about screening or unsupportive of its potential benefits. Instead, their consideration of the perceived necessity, legitimacy and utility of this screening shaped their decision not to take part.

**Patient or Public Contribution:**

The SAFER programme is guided by four patient and carer representatives. The representatives are embedded within the team (e.g., one is a co‐applicant, another sits on the programme steering committee) and by participating in regular meetings advise on all aspects of the design, management and delivery of the programme, including engaging with interpreting and disseminating the findings. For the qualitative workstream, we established a supplementary patient and public involvement group with whom we regularly consult about research design questions.

## 1 INTRODUCTION

Screening programmes have a clear ambition: to scrutinise asymptomatic individuals to determine the likely presence or absence of a condition, the early treatment of which at scale will reduce population morbidity and untimely mortality.^1^ Population benefit from screening is expected to be maximised when participation is high. Many screening programmes, therefore, set explicit participation targets (e.g., Australian Institute of Health and Welfare; Richards).^2^, ^3^ which persist despite recognition of (and in tension with) the importance of informed choice in screening participation.^4^ Correspondingly, there is expansive literature on efforts to improve the uptake of screening and reduce inequalities in access.^5^, ^6^, ^7^ Screening participation is encouraged by societal imperatives about individual responsibility and the priority of health, which make participating a ‘good’ thing to do.^8^ The nonparticipant is often presented as subject to ‘barriers’ to participation and as missing out on the advantage of screening, with little known about those who may *choose* not to take part. In this article, we explore the reasons for actively declining to participate in atrial fibrillation (AF) screening in England, offered as part of a large national trial.

### 1.1 The social context of screening

Sociological work in the 1990s and 2000s showed how modern medical and epidemiological advances enabling the identification and treatment of ‘risk‐factors’ of ill‐health, interplaying with societal expectations about prioritising good health and taking personal responsibility, made mitigating health‐related risks an important and moral task for individuals.^9^ Screening, consequently, offers an enticing promise to prevent future ill health by identifying and addressing potential problems in the present, while also reassuring the participant that they are acting responsibly and doing the ‘right thing’.^8^ Studies of screening participation have shown that those who take part typically do so because it is a ‘healthy choice and […] part of a wider portfolio of being healthy’^10^ and highlight the ‘moral obligation’ to engage in screening (e.g., Howson).^11^ Underlying the presumption of participation is the risk that those who do not participate in screening are stigmatised as ‘irrational, self‐deluding and irresponsible’,^12^ ‘wilfully ignorant’ or ‘non‐compliant’.^13^

Of course, not everyone takes part in screening when invited, and nonparticipation is socially patterned. Associations between income‐related inequalities and low participation in screening programmes are found internationally (e.g., Devaux; Quintal & Antunes).^14^, ^15^ There is strong evidence that lower attendance in cancer screening is associated with sociodemographic factors including low socioeconomic status; non‐White ethnicity; having a learning disability and, to some extent, age.^16^ Seen across screening programmes, these associations mean that ‘people at higher risk of the condition being screened are less likely to participate’.^17^ This is especially significant for providers who seek to maximise informed screening participation to realise population health benefits while having legal duties to reduce inequalities in screening access (e.g., Public Health England).^17^

Screening research attention has therefore focused on understanding who does (and does not) attend screening, and investigating factors that may predict screening attendance and nonattendance, with psychological research at the vanguard.^8^ Nonparticipants can be categorised as ‘passive’ decliners, who mean to participate but ultimately do not, or who have not received or engaged with an invitation, and ‘active’ decliners, who have chosen not to participate (e.g., von Wagner).^18^ Identified barriers to cancer screening participation include perceptions of the screened condition or the screening itself (including considering it stigmatising, fear or lack of knowledge of the condition or belief in screening efficacy, low health literacy^16^ and practical challenges such as travel difficulties, caring responsibilities and being too busy (e.g., Ali et al.).^19^ This literature helpfully describes and explains trends in screening participation. However, by focusing on identifying (and seeking to resolve) barriers to participation, it often inadvertently risks blaming the nonparticipant, perhaps because typically the underlying aim of such research is to maximise screening uptake.^20^

It seems possible, therefore that the literature on pragmatic and quantifiable ‘barriers’ to nonparticipation does not address all the reasons why individuals may *choose* not to take part. This literature may occlude, for example, reasons to do with the screening programme itself^20^, ^21^ and may oversimplify a complex decision‐making process in which individuals may see screening as positive but may not find that a sufficient driver for participating (e.g., Oscarsson et al.).^22^ Sociological work has shown that an individual's sense of obligation to participate in screening can be moderated by their assessment of the degree of personal applicability and personal benefit, whether because they resist discourses that they are at risk of the screening condition or because they do not recognise themselves as possible candidates for it (e.g., Armstrong; Bikker et al.).^10^, ^23^ These accounts have helped to shape insights into why some people do not participate in screening when it is offered to them. In this study, we aim to refine our understanding of screening nonparticipation and to further characterise the influences that drive people's decisions to actively decline an offer of screening, through the accounts of those who chose not to undergo screening for AF.

### 1.2 AF screening

AF is a common, often asymptomatic, heart arrhythmia that increases in prevalence with age.^24^ Having AF increases the risk of stroke fivefold,^25^ although this risk can be effectively reduced through anticoagulant medication.^26^ AF detection approaches include portable, simple‐to‐use technology, such as handheld and wearable electrocardiogram (ECG) devices, and no‐tech pulse palpation. Consequently, AF is considered a possible candidate for screening.^27^ In the United Kingdom, screening is softly endorsed by NHS England's aim for increased AF detection,^28^ achieved, for example, by clinicians performing pulse checks at routine primary care appointments for those aged over 65, or local schemes using mobile‐health ECG devices supported by the Academic Health Science Network, an NHS England and industry partnership.^29^ While such opportunistic screening occurs, national screening bodies have not recommended the establishment of systematic AF screening programmes due to insufficient evidence to date on effectiveness and cost‐effectiveness.^30^, ^31^

While the expectation of participation in well‐established population‐based screening programmes may be fortified by societal concern about and fear of the disease (e.g., cancer)^32^ or expectancies of responsible parenthood (pregnancy and neonatal screening),^33^ AF screening is a novel offer for a condition that is not widely known by the public.^34^ This, then, is an opportunity to explore how people respond to an invitation to engage with screening and account for their decision, outside of established screening programmes that may be considered routine.^8^ In particular, it provides a more neutral context in which to focus on personal choice not to participate, and the reasons people draw on when accounting for their decision to decline (cf., McCaffery et al.).^35^

#### 1.2.1 The trial

SAFER (Screening for Atrial Fibrillation using ECG to Reduce stroke) (https://www.safer.phpc.cam.ac.uk/) is a publicly‐funded trial aiming to inform clinical guidelines and national screening recommendations.^36^ It requires participants to use a handheld ECG device (www.zenicor.com) four times a day for between 1 and 4 weeks. The trial is ongoing, with two feasibility phases and an internal pilot trial completed. Potential SAFER participants are drawn from participating general practitioner (GP) practices in England. Eligible patients are aged over 65 (first feasibility phase) or 70 (all subsequent phases). SAFER participation involves a two‐stage consent process: first, to the study, and second to screening. Not all SAFER participants are invited to screening: while all feasibility phase participants were invited, pilot phase participants were randomised by GP practice to control or screening intervention group.

The initial participant information sheet (PIS) introduces AF as a ‘heart condition that causes an irregular heartbeat. It affects [up to/over] 1 in 10 people over the age of [65/70] but does not necessarily cause symptoms. Having AF increases the risk of having a stroke 5‐fold, but treatment with medication can significantly lower this risk as well as lowering your risk of having a heart attack’ (variation reflects earlier versions of the PIS). The PIS explains that participation will involve consenting to sharing health data and potentially being invited to screening and associated studies (qualitative interviews or questionnaires about the screening).

Participants who consent to participate and are included in the screening arm are invited to the screening. In a second PIS, participants are informed about screening practicalities, benefits (if found to have AF, starting treatment would reduce the risk of stroke, heart attack and potentially dementia), potential risks (screening‐induced anxiety, and for those found to have AF, bleeding‐related medication side‐effects and insurance implications) and the reliability of the test (low chance of incorrect diagnosis of AF, possible chance of missing diagnosis).

#### 1.2.2 Choosing not to take part in SAFER: Early findings

In previous work, we found that SAFER participants considered AF screening to be legitimate and worthwhile. They considered their nonparticipating peers to be deviating from good preventative practice and to be putting themselves at risk, characterising them as ‘uninformed, indolent, irresponsible, wilfully ignorant, or gratuitously anxious’.^34^ However, our scoping study of nonparticipation showed that those not taking part were neither uninformed nor wilfully ignorant: they recognised the value of screening and thought it worth overcoming inconveniences to take part.^37^ Moreover, for SAFER participants, engaging in the screening seemed to be almost a ‘non‐decision’ because it was so clearly the ‘right’ thing to do.^34^ This suggested that to *not* participate, to not comply, may be a decision. This interpretation of nonparticipation fits awkwardly with the idea of the non‐agentic‐screening‐avoider as portrayed by participants and in the ‘barriers’ screening literature. If nonparticipation was a choice, though, we were unsure why: exploring this was beyond the scope of our initial study. It is this topic we address in this article.

## 2 METHODS

### 2.1 Data collection

The SAFER trial includes an embedded qualitative research programme that contributes to ‘addressing the overall aims of SAFER, namely, to consider the feasibility, harms, effectiveness and cost‐effectiveness of a national screening programme for AF in primary care’.^38^

**Figure 1:**
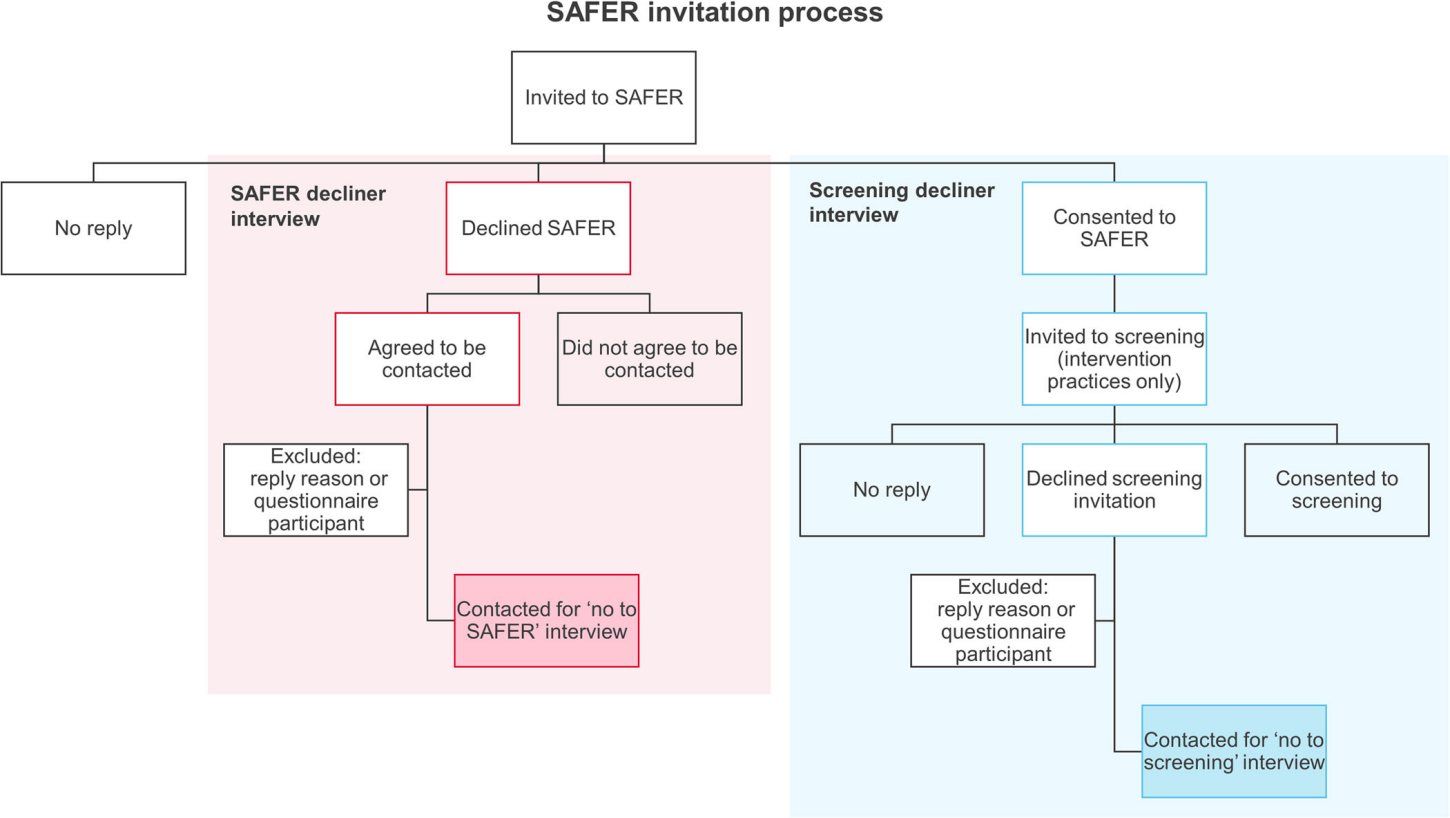
Screening for Atrial Fibrillation with ECG to Reduce stroke (SAFER) interview identification process.

We conducted interviews with people invited to SAFER in the feasibility phases and the internal pilot trial. We sampled participants from (a) those who had declined an invitation to participate in SAFER (‘SAFER decliners’) and (b) those who consented to take part in SAFER but had declined an invitation to participate in AF screening (‘screening decliners’). We included both participant groups as our scoping study of nonparticipation showed that people declining to participate in SAFER typically did so because they did not want to participate in screening.^37^ We identified potential interview participants from reply slips, which asked participants to state reasons for declining study or screening participation. Except where they stated a reason that met our exclusion criteria (see Box 1 for criteria) all participants were classified as ‘eligible decliners’. Figure 1 provides a diagram of this process.

Box 1 **Interview exclusion criteria**

We excluded potentially eligible interviewees who reported:

○ being ineligible for SAFER (e.g., moving out of area)
○ issues unrelated to screening (e.g., concern about data protection)
○ significant distressing life events (e.g., recent bereavement)

- *or* did not agree to be contacted by a researcher
- *or* were randomised to receive a SAFER health questionnaire, to avoid study burden.

In the feasibility phases, we approached all eligible decliners. In the pilot trial, we approached all eligible decliners from six consecutive practices. Our recruitment method differed by decliner group. We telephoned SAFER decliners to provide more information about the interview. If they were interested, we booked an interview and sent them an interview pack (including PIS and consent form). Screening decliners were first sent an interview pack and those who returned a positive reply slip were then booked in for an interview. We interviewed 50 participants across the two feasibility phases and the pilot trial. Table 1 lists the interviewees' characteristics.

**Table 1:** Sociodemographics of interviewees and data collection phase.

	Total	SAFER decliners	Screening decliners
Total	50	29	21
Phase			
Feasibility 1	24	12	12
Feasibility 2	2	0	2
Pilot	24	17	7
Gender			
Female	29	16	13
Male	21	13	8
Decile of practice deprivation (10 = least deprived)^a^			
10	7	3	4
9	5	3	2
8	21	14	7
7	1	0	1
6	14	8	6
5	2	1	1
4–1	0	0	0

Abbreviation: SAFER, Screening for Atrial Fibrillation with ECG to Reduce stroke.

^a^ From Public Health England^39^ National General Practice Profiles, using the English Indices of Deprivation to calculate the Index of Multiple Deprivation: ‘an overall measure of deprivation experienced by people living in an area’.^40^

SH or GT conducted each interview by telephone and took verbal recorded consent at the start. The interviews were audio recorded, lasted approximately 30 min each and were conducted between 2019 and 2021. The interviews were semi‐structured and guided by a flexible interview schedule (see Box 2).

Box 2 **Summary of interview topic guide**

Interviewees were asked about:

- The trial/screening invitation.
- Reasons for not participating.
- Screening in general, including prior participation in NHS screening programmes.
- The SAFER trial.
- Impact of the COVID‐19 pandemic *(feasibility 2 phase onwards)*.

The trial, including the qualitative programme, has been approved by the London‐Central NHS Research Ethics Committee (reference numbers 18/LO/2066 and 19/LO/1597).

### 2.2 Analysis

The interview recordings were professionally transcribed verbatim. Taking a sociologically informed approach, we used a reflexive thematic analysis method^41^ to explore the reasons why people did not participate in AF screening. We assumed that interviewees were knowledgeable about the reasons why they had not participated in AF screening and that their accounts, while not necessarily ‘true’ descriptions, could provide us with insight into their motives for not participating. SH led the analysis, conducting the coding initially on paper and then supported by the software NVivo 12. The codes were generated inductively from topics raised by interviewees and deductively from the interview schedule: starting with prevalent practical reasons for nonparticipation in participants' accounts, we subsequently identified nascent references to the necessity, utility, and relevance of the screening and explored again the data set for these concepts and how they interacted with the practical reasons already coded. Our analysis and synthesis process was informed by our scoping study of nonparticipation,^37^ discussions with the wider authorship group and reference to social science and health literature about screening.

Quotations are followed by interview ID number (1‐50), practice code (A‐R) and declining phase (‘SAFER’ or ‘screening’).

## 3 RESULTS

Interviewees offered many practical reasons and concerns about the screening to explain their decision not to participate. Throughout their accounts, interviewees also explored considerations about the necessity, legitimacy and utility of the screening on offer. We argue that these considerations were instrumental in how interviewees framed the merits of AF screening and the practical burden of participating, shaping their decision not to participate.

### 3.1 Practical issues and screening and outcome concerns

Misunderstandings about what the screening involved made participation for some impractical or undesirable, such as expecting to have to travel to be screened or to undergo an invasive screening test, while a few had misread exclusion criteria. Others found the invitation had arrived at an inopportune moment, exacerbated by perceptions of a demanding screening programme. Those with busy schedules (caring responsibilities, work) raised concerns about how the screening intensity and duration could negatively impact these commitments, while interviewees with long‐term health conditions or frailty explained that participating would be ‘too much’ on top of everything else. Collectively, these accounts implied that the screening asked unduly of their time:

I: what made you think that actually, like, this trial just isn't for me?

R: It was the extra time I would have to put in, while probably not feeling very well at the same time, if you see what I mean? [I: Yes.] And the last thing I want to do is feel worse than I do already. (25Q_SAFER)

I thought there's no day with my lifestyle that I'm going to be able to stop and doing something every four hours or whatever it was, I can't remember now. I thought I might be on the plane, I might be giving a lecture or something else and I can't stop to do whatever it was. (49A_SAFER)

Interviewees concerned about doing the screening ‘right’ were also put off participating. They were worried about ‘pressing buttons and remembering the sequence in which I had to press them’ (1O_Screening), forgetting to routinely use the device, or inadvertently providing incorrect traces. These issues exercised interviewees because they felt they could disrupt the research and were significant enough for some to frame it as breaking a commitment inherent to agreeing to participate: You've got to finish a screening, you can't stop and get halfway through, you've got to finish your screening otherwise you really aren't going to find anything out (10F_SAFER).

Relatedly, some expected that taking part would be a ‘stressful process’ (32D_Screening), whether because using the device could ‘trigger anxiety’ (35A_Screening) or from waiting for and potentially receiving a positive result and the consequences of it. While interviewees sometimes spoke disparagingly about these concerns, presenting them as irrational because they stopped them from participating, the reasons they attributed to their screening anxiety often described significant healthcare experiences:

I have PTSD from medical experience that I've had […] And the thought of having to hold something or be monitored for four times a day, my whole body just goes, I can't do that, I can feel the tension rising. (41J_Screening)

Interviewees were also discouraged by the consequences of potentially receiving a positive result, including travel insurance concerns and having to take anticoagulant medication. Here, contextualised by their own current health problems or the experience of others, interviewees raised well‐recognised apprehensions about potential bleeding side effects and dietary interactions (e.g., Borg Xuereb et al.).^42^

While this is a comprehensive summary of the *reasons* interviewees gave, these justifications for nonparticipation do not represent the totality of their accounts. We additionally identified small and dispersed, but repeated, interviewee suggestions about the necessity, legitimacy and utility of the screening that collectively framed their decision not to participate.

### 3.2 Positioning nonparticipation: Necessity, legitimacy and utility of screening

#### 3.2.1 Screening necessity: Alternative routes to health

Few interviewees knew about AF and, in this void, assumed it was like other heart‐related conditions. Interviewees often expected initiatives to address these (such as receiving medical care for related conditions or engaging in a healthy lifestyle) would also be protective against developing AF and a potential subsequent stroke, thereby making participation unnecessary. Some interviewees also inferred AF was a hereditary condition and that as there were ‘no heart problems in the family’ (29C_Screening) or because their older relatives had enjoyed long lives, they did not need to participate. AF was also often presumed to always involve palpable symptoms. Many interviewees therefore discounted the health benefits of screening participation as they had not experienced these symptoms, or because they anticipated that if they later experienced them, they would proactively seek healthcare.

I haven't heard of [AF]. I knew [the screening] was something to do with the heart, but I thought, well, mine's sound so I won't bother. (38J_SAFER)

[T]he doctor knows all about me already. I go regularly for check‐ups and I have my blood pressure done and cholesterol and all that and I'm on medication for raised blood pressure, but I don't have any problems with my heart at all as far as I know. So I just couldn't see how a screening would be…well, I felt it would be a waste of time really, for me. (19A_Screening)

Prior or underlying health conditions meant some interviewees did recognise they were at risk of a stroke. For some, the medication and medical surveillance they already received devalued the screening offer. For others, while their experience made the screening pertinent, it was not a priority in the context of other risk factors, or the work associated with their condition(s).

I thought probably I needed to do it but I couldn't be bothered because I'm already taking things to reduce the risk of stroke anyway. So I thought well…just hope I don't get another stroke. […] If that's the price I have to pay for the atrial fibrillation. (1O_Screening)

#### 3.2.2 Screening legitimacy: The voluntary and unofficial nature of SAFER

SAFER is a research programme and this underscored many interviewee accounts. Interviewees were supportive of research and research participation, which they typically positioned as a way of showing gratitude towards the healthcare service (especially for those interviewed during the COVID‐19 pandemic). However, SAFER being research also meant that participation (and importantly, the work of participating) was seen as voluntary. Altruism was equally fostered by participating in an interview, and (for screening decliners) by agreeing to share their data within the SAFER study.

I would have liked to have taken part basically to support our wonderful practice. To support…I'd like to feel I'm being helpful to other people, and that I could be of some use, without…I was going to say, without being too selfish. I am. And I think it's probably good that these things are taking place. And yes, except I just didn't want to do that bit of it [the screening], but I am quite happy doing the other [being part of the trial]. (35A_Screening)

Recognising this screening was part of a trial, interviewees also sometimes unfavourably presented it as experimental, with concomitant concerns about the robustness of the test, the validity of the result, and whether treatment would be offered following a positive result. This was contrasted with the established nature and status of national screening programmes. For some, these differences undermined the presentation of SAFER as legitimate screening, and meant that they would consider participating if it were ‘authorised’ (46B_Screening):

I think, because they've [national screening programmes] been ongoing for such a long time, so it's like okay, you know, it's worth doing because who wants to get breast cancer or, you know, to not have it diagnosed early, and who wants to have bowel cancer, and here's a relatively easy way of, you know…of, kind of, keeping things in check hopefully. Although I know it's not fool proof, but…so I haven't really had a problem, so it was more duty to myself. But this was different [AF screening] because it's new. It's an un…a lot of unknowns. (26Q_SAFER)

If it was offered to me as a screening it definitely works as a screening and will lead to some medication that will definitely prevent something happening, then clearly I would certainly somehow have found the time to do it all. (36P_Screening)

#### 3.2.3 Screening utility: Ageing, dying, and too much ‘poking around’

Interviewees' age, and for some, their recognition that they would die in the not‐too‐distant future justified not taking part in the screening. Interviewees linked this to pragmatic concerns: being older explained their poor memory which could compromise adhering to the screening schedule, made the work of participating harder and their time more precious to give, lessened their research contribution because they would soon die, and exacerbated anxiety about having a positive result. Older age also challenged the utility of the screening: however ‘healthy’ interviewees were, they recognised being older made it more likely that they would have AF or experience ill health as ‘part of the territory of being old’ (6O_Screening). Often, interviewees commented that the screening would be better targeted at younger age groups who were presumed to be better beneficiaries of the screening opportunity (and implied conversely that they would not utilise the benefit because they would die soon). A few interviewees were more direct, explaining that the screening was irrelevant because it would not change the immutable fact of their imminent death: ‘there's no way you're going to escape’ (3G_SAFER). Screening could also change how their remaining life, dying or death might occur. Bound to the preventative hope that screening offered the potential to extend life or delay ill health, interviewees indirectly presented screening as an unwelcome disruptive process to their remaining life and dying.

I should think by now I've reached my sell by date so, you know, it doesn't worry me. I've got to die of some…everybody has to die of something, and well I was 87 last week so, you know, it doesn't worry me in any way. And I…but I think it's too late for somebody like me to take part in research, yeah. I mean, if I've got anything, I don't know if I have got anything, you know, it's just the end of life, isn't it. (16F_SAFER)

Well I just thought I don't know what's this poking about and doing things at my age, 93. Oh no, leave it to the young ones. (45F_SAFER)

Interviewees were ambivalent about the principle of screening within the context of SAFER and implicitly challenged the rhetoric of beneficial early AF diagnosis. While screening was a ‘good thing’ or a ‘good idea’, and interviewees explained they had previously engaged in screening programmes, they struggled with talking about the place (and problem) of AF screening in their own lives. Interviewees voiced concerns that participating in screening could undesirably unearth issues that they would not otherwise have had to address at the time, and inferred, sometimes explicitly, that screening would be disruptive to their lives. This was most tangibly presented through concerns that participating in screening risked being ‘sucked into a medical issue’ (32D_Screening), particularly if they subsequently received a positive result and were expected to engage in further testing and treatment. Concerned that they would have little agency to resist when ‘caught in, immersed in a medical process’ (17M_Screening) of whose value they were unsure, not participating could be a pragmatic decision, although one that some were apologetic about:

Because if I had atrial fibrillation then it's got to be treated. Then you are in a spot…oh this sounds so ungrateful. [I: No.] You're in a spiral of then, you're caught up in spiral of health issues which I'm pleased could be prevented, but needn't be started in the first place. (35A_Screening)

Rarely, interviewees went further to draw explicit parallels between the SAFER screening offer and wider concerns about medicalisation and screening, such as in derogatory comments about the societal priority given to preventative health or overzealous health risk‐mitigation strategies such as statin prescribing. Instead, interviewees' hesitation about screening was interwoven with other reasons for nonparticipation. The screening programme was costly to participate in: interviewees anticipated it to be demanding, anxiety‐inducing for some, disruptive and time‐consuming, requiring effort to do it ‘right’. Meeting these sacrifices offered the promise of advantageous early diagnosis and intervention; a promise compromised by their questioning of the potential benefit. While their assessment of the screening was individual, collectively their view of it was coloured by their prevalent identification of the screening as research and thus unofficial and optional, and in the context of their lives and their life stage, as of little relevance:

I recognise that [screening is] a good thing and the right thing to do […].…the whole [screening] experience is quite stressful. […] And to me that is a real risk, you know, that you're inviting problems into your life which you would not have if you weren't screened. So if there was a balance as to whether…where the benefit lies, you know, is it worth doing, bearing all these things in mind, or not, usually I think the benefit outweighs the disadvantages. So that's usually my attitude to being screened for anything, but it is a stressful process and requires thought. So when you invited me to take part in this study, I thought, oh God, you know, surely I haven't got this atrial fibrillation, I've got no symptoms. So I thought, well, I have had ECGs in the past, they've been fine, hopefully I'll be alright, yes, I should do it. And then the balance shifted when I found out how relatively onerous, if I can use that word, the process was going to be. So at that point I thought, no, this is a step too far, and I declined. (32D_Screening)

## 4 DISCUSSION

Challenges to the necessity, legitimacy and utility of AF screening were integral to interviewees' choice not to participate, despite their broad support for screening. These were voiced through their framing of, and concerns about the practical tasks of participating, including misunderstanding instructions, travel problems, being too busy (including caring for others), health problems, bad timing, anxiety and, more rarely, the implications of the ‘spiral’ of treatment for a positive result: issues well‐recognised in the literature (e.g., Hope et al.; Lin et al.; McCaffery et al.; McCoyd; Reid et al.; Young et al.).^35^, ^43^, ^44^, ^45^, ^46^, ^47^

In opposition to prevalent depictions of nonscreeners as at fault in some manner, interviewees showed considerable consideration in their choice not to participate, comprehensively reviewing the screening from test through to the result, as others have found.^48^ Interviewees made a detailed assessment of their risk of having AF, interpreting accepted preventative medicine messaging through the prism of their own lives (cf., Davison et al. 1991),^49^ replicating the decisions of nonscreeners elsewhere (e.g., Aasbø et al.; Nielsen et al.).^50^, ^51^ From the evidence presented to them, interviewees accepted that AF was a serious condition and, referencing appreciation of preventative healthcare, thought screening a useful opportunity to address this risk for others. Their own self‐view as healthy and able to address their AF risk if symptoms arose, or mediate this risk through existing health practices, matches nonparticipation decisions in other screening programmes (e.g., Chapple et al.; Chien et al.).^52^, ^53^ In this context, echoing concerns of other nonparticipants across screening programmes (e.g., Berg‐Beckhoff et al.; Maclean et al.),^54^, ^55^ engaging in screening risked ‘making’ AF and opening Pandora's box of ‘trouble’. This was not irrational: testing for a condition they did not think they had (but that they recognised could be identified by screening) would create unhelpful awareness of problems in their body and disrupt their health.^56^ Moreover, their acceptance that they would die ‘when their time has come’ as Davison et al.^57^ found (and since found in screening^50^), exposed the mirage of screening and preventative health practice in halting death.^58^ Together these factors rebalanced perspectives to place more weight on the effort of participating against the benefit of addressing future ill health.

Interviewees' considerations about not taking part, and their deviation from what could be considered ‘acceptable’ behaviour, offer insights into the possible limits of screening obligation. Interviewees' accounts were different from those who took part in AF screening who sometimes struggled to articulate exactly why they did so, as inherently obvious as it was to engage in screening.^34^ This echoes Polak and Green's^59^ preventative medication decision‐making study: while statin‐takers presented ‘no‐choice’ but to take them, nontakers stressed their need to ‘think about it’, prioritising their concerns about medication side‐effects to explain their decision to deviate from a clinician‐endorsed approach. Across both studies, those taking part and those not doing so drew on similar knowledge and evidence to decide. They recognised the ‘right’ thing to do (to take statins, to engage in screening) and taking the contrary approach involved careful justification. Interviewees achieved this by assessing the participation risks and benefits within the context of their ageing lives and the perceived limited legitimacy of an experimental screening programme. Other studies of screening refusal similarly show how those not participating use their own experience to assess their risk, with such decisions based on an interplay of personal views, values and one's social context.^50^, ^60^, ^61^ Together, this research suggests a limit to the sense one ‘ought’ to engage in preventative health practices, notably when the personal legitimacy of the screening offer is not recognised. Bikker et al.^10^ study of colorectal screening attenders and nonattenders reiterate this: only attenders were motivated to participate by the preventative messaging of screening, so that ‘the moral structure that underpins the new public health can be witnessed practically in the way in which *those who see themselves as candidates for screening* embrace wider positive health practices’ (p. 18, our italics).

Perceptions of reduced legitimacy inherent within the AF screening invitation—as unofficial, experimental, voluntary work—were significant for enabling interviewees to choose not to participate. Elton^62^ argues that the nature of national screening invitations encourages participation. They carry an inducement to take part because the inviter is a health professional: someone who has greater authority than the patient clinically and who they can expect ‘would not invite them to participate in an intervention unless [the clinician] expected that intervention would be of benefit’.^62^ The AF screening invitation, while from the interviewee's GP practice, was also recognised to be part of a research study, moderating inducements to participate. Nonparticipation research has similarly shown this, whether as a novel screening programme that inhibited obligation,^35^ or with follow‐up prenatal screening that no longer feels routine and where participants feel the authoritative knowledge of clinicians is challenged such that they can more freely decide about participating.^45^, ^48^

### 4.1 Limitations

It should be expected that there will be socioeconomic and cultural explanatory trends in reasons for nonparticipation^63^ which we did not explore: we had very limited information about interviewee socioeconomic, cultural and demographic characteristics. Furthermore, interviewees were less deprived by practice deprivation status compared to the population of England, and it seems likely that those who responded positively to our interview request considered that they had a ‘legitimate’ reason for not wanting to participate and sufficient social capital to justify a decision they recognised to be potentially socially deviant, or did not perceive SAFER activities as screening, potentially limiting the contribution of our findings.

### 4.2 Future research

Interviewees identified many costs to participating in AF screening. While their views differ from those who chose to participate in SAFER and who presented the screening decision as straightforward and easy,^34^ it seems likely that it is not that the costs were different but that the burden of them was differently felt.^64^ Identifying these nuances deepens our awareness that screening participation can be harmful and is never cost‐free. Interviewees' anticipated anxiety about AF screening explained through reference to their previous experiences of screening attendance, for example, highlights the psychological harms that are borne (and tolerated) by screening participants. Further studies are needed to investigate the harms of participating in AF screening, including the types and scope of harm, and how they could be minimised.

Research too could explore how the sense participants feel they ought to take part in screening interacts with ageing and how screening is presented. It seems plausible, for example, that if AF screening were to enjoy the benefits of widespread healthcare provider endorsement and publicity associated with being a national programme, many interviewees would have participated. Their underlying belief that screening was good was a helpful prime for this new programme but was challenged by their perception of it as optional and their ‘fatalism’ towards their health in old age and proximate death. Screening programmes seeking to provide sufficient information for invitees to make an informed choice about participation should consider how the evidence about the benefits of screening for older adults is presented, paying particular attention to the wider challenges about the necessity, legitimacy, and utility of AF screening. Embedding qualitative programmes alongside screening trials^38^ or co‐designing screening programmes^65^ may help to ensure screening programmes address the needs and priorities of potential participants and empower their decision‐making about participating. However, values about screening are ultimately made within the context of an individual's lifeworld, and recognising participants' agency to decide (not) to take part is necessary for understanding screening participation.^66^

### 4.3 Conclusion

Using a sociologically informed approach, we sought to understand nonparticipation from the perspective that individuals may choose not to take part in screening and that they will have a reason for this that makes sense to them. Our research shows the contribution of nonscreening participant voices for understanding the social significance of screening and reinforces that not participating in screening can be a reasoned decision. Nonscreeners were not resistant to the perceived obligation of engaging in health‐related behaviour, unaware of screening rhetoric or unsupportive of its benefits. Instead, their decision not to participate was affected by their individual weightings of the perceived necessity, legitimacy, and utility of AF screening.

## AUTHOR CONTRIBUTIONS

**Sarah Hoare**: Methodology, formal analysis; investigation; data curation; writing—original draft; writing—review and editing; project administration. **Gwilym P. A. Thomas**: Formal analysis; investigation; data curation; writing—review and editing; project administration. **Alison Powell**: Writing—review and editing. **Natalie Armstrong**: Conceptualisation; writing—review and editing; funding acquisition. **Jonathan Mant**: Conceptualisation; writing—review and editing; funding acquisition. **Jenni Burt**: Conceptualisation; methodology; writing—review and editing; supervision; funding acquisition.

## CONFLICTS OF INTEREST STATEMENT

Natalie Armstrong is a member of the UK National Screening Committee. Jonathan Mant has received honoraria from BMS/Pfizer. The remaining authors declare no conflict of interest.

## ETHICS STATEMENT

The trial has been approved by the London‐Central NHS Research Ethics Committee (reference numbers 18/LO/2066 and 19/LO/1597).

## Data Availability

Data are available upon reasonable request. Please email the PI Jenni Burt (jenni.burt@thisinstitute.cam.ac.uk) for details.
